# Investigation of the inverse problem for the Arrhenius equation using the example of thermal degradation of spongin-based scaffolds

**DOI:** 10.1038/s41598-023-29565-1

**Published:** 2023-02-14

**Authors:** Sonia Żółtowska, Michał Ciałkowski, Krzysztof Alejski, Teofil Jesionowski

**Affiliations:** 1 Faculty of Chemical Technology, Institute of Chemical Technology and Engineering, Poznan University of Technology, Berdychowo 4, 60965 Poznan, Poland; 2 Faculty of Environmental Engineering and Energy, Institute of Thermal Engineering, Poznan University of Technology, Piotrowo 3, 60965 Poznan, Poland

**Keywords:** Chemistry, Energy science and technology, Materials science, Mathematics and computing

## Abstract

A mathematical description of the thermal degradation of spongin-based scaffolds is given. The Arrhenius integral was evaluated using the inverse problem approach, in which the unknown values were the activation energy *E*_*A*_, the pre-exponential factor *A*, and the model function *f*(α) characterizing the physical process. The form of *f*(α) was determined and the values of the parameters *E*_*A*_, *A* and *T*_*S*_ were evaluated in detail. Moreover, the function *f*(α) assessed in this study was compared with classical solid-state model functions. Finally, the mean square minimization approach was used to solve the inverse problem with unknown function *f*(α) and pre-exponential constant *A*. Likewise, the approximation of *f*(α) with 6th- and 7th-degree polynomials was used to obtain numerical values of *E*_*A*_ and *A*. This study evaluated the inverse problem approach for the Arrhenius equation. These investigations provide new insight into the description of the thermal degradation of spongin-based scaffolds.

## Introduction

The thermal reactions, especially thermal degradation and pyrolysis, of any material are essential for the design of industrial processes^1–4^. Knowledge of the thermal behavior of solids provides key information about the proper conditions of their synthesis and optimal conditions for their utilization. Hence, for fifty years, techniques for investigating the kinetics of thermal degradation of any solid, of either synthetic or natural origin, have been extensively developed in modern chemical engineering^3,5–15^.

The determination of kinetic parameters, including activation energy and pre-exponential factor, is based on thermogravimetric analysis^4,16,17^. In this approach, the kinetic parameters are evaluated using reactions that produce a mass change or change in enthalpy. An advantage is that a small amount of material is used for the measurement—not exceeding 20 mg. The data showing mass loss as a function of temperature and time are converted to a standardized form called the degree of conversion α, ranging from 0 to 1. This is a measure of the progress of the reaction as a function of time or temperature^18,19^. Within this method, two alternative techniques have been developed. In the first, yield–time measurements are performed for a reactant maintained at a constant temperature. In the second technique, the sample is heated in accordance with a programmed increase in temperature, and the mass changes are measured as a function of time or temperature. Currently, this approach is the subject of detailed investigation because it provides more reliable and practically valuable results. It has been successfully used to determine the kinetic parameters of various lignocellulose-based materials such as rice husks^15,20–22^, wood waste^23^, and rape straw and stalks^7,22,24^, as well as proteins^8,25–27^.

When discussing approaches to kinetic investigation, it is essential to consider the mathematical background. All of the methods mentioned above assume the Arrhenius equation as a keystone for further evaluating the kinetic parameters^28,29^. The integration of the Arrhenius equation over time as a function of temperature results in a temperature integral, which does not have an analytical solution^30,31^. Nevertheless, many published studies describe the application of the integral approach, using both model-fitting and model-free methods, evaluating the activation energy and pre-exponential factor for thermal degradation and pyrolysis processes^3,5,7,11,32–37^. Reviews of work in this area have also appeared^14,38–41^. These publications reveal several different approaches to calculating the temperature integral. A significant study indicating the most accurate approximations for temperature integrals was published by Orfao^39^. Numerical methods are not often applied to evaluate the thermal degradation kinetics^42–45^ and other physicochemical processes^46^. The approaches already described vary significantly; consequently, finding the optimal strategy for calculating the thermal degradation kinetics of any material is still a challenging task.

On the other hand, only one paper (based on our literature review) contains some criticism relating to the work already published on the mechanism and kinetics of thermal reactions, principally pyrolysis^41^. In any case, these publications are more than ten years old. In the last decade, investigations dealing with kinetics and thermal degradation mechanisms have mostly adopted insufficiently consistent approaches to enable the comparison of the numerical values calculated for various materials. Some general concepts and ideas can introduce scientific order into the available information. However, the majority of works based on thermogravimetric analysis remain unrelated to theoretical principles that can be accepted in chemistry.

An analysis of the literature related to the determination of the real transformation function *f*(α) in the Arrhenius equation (thermal degradation process) revealed the possibility of presenting the function *f*(α) in the form of a polynomial equation. Therefore, for the process of thermal degradation, the determination of the polynomial coefficients, measured for the temperature function *T* = *T*(α), taking into account different rates of the degradation process, is an inverse problem.

The motivation for seeking a new, practical approach to the evaluation of kinetics came from our previous study, in which a classical numerical approach was applied to evaluate, for the first time, the thermal degradation behavior of spongin-based scaffolds^47^, using a model-fitting approach (the Coats–Redfern method) and the model-free iso-conversional Friedman, Kissinger–Akahira–Sunose (KAS) and Ozawa–Flynn–Wall (OFW) methods. The results of this work encouraged us to attempt to give a formal description of the process of thermal degradation of spongin-based scaffolds.

Consequently, this study includes a formal, mathematical analysis of the thermal degradation of spongin-based scaffolds. The Arrhenius integral is evaluated using the inverse problem approach, in which the unknown values are the activation energy *E*_*A*_ and pre-exponential factor *A*, and the model function *f*(α) characterizing the physical process is determined based on the temperature function *T* = *T*(α) (where α is the degree of conversion). The investigation was carried out using the example of thermal degradation of spongin-based skeletons.

The explicit form of the model function *f*(α) is not known. Thus, the fundamental problem was first to determine the class of functions to which the model function belongs, and then to determine an approximation of the function *f*(α) parametrically. Next, the function *f*(α) will be presented as a polynomial of degree *n*. This is a novel approach not described in the existing subject literature.

## Experimental part

### Kinetics of thermal degradation of spongin-based skeletons

The kinetic equation of the process of thermal degradation of spongin was formulated in relation to the change in mass of the sample subjected to degradation, *m*_*α*_* – m*_*f*_:

1 $$r=-\frac{d({m}_{\alpha }-{m}_{f})}{dt}=k\left(T\right)F\left({m}_{\alpha }-{m}_{f}\right)$$

2 $$k\left(T\right)=Bexp\left(\frac{-{E}_{A}}{RT}\right)$$

where *t* denotes time (min), α is the degree of conversion, *F*(*m*_*α*_* – m*_*f*_) is the kinetic part of the equation related to the mass of reactant taking part in the reaction, *m*_*α*_ and *m* are the masses of the sample measured for a specified α and in the final stage respectively, *k*(*T*) is the kinetic rate constant, *B* is the pre-exponential factor, *E*_*A*_ (kJ/mol) is the activation energy, *T* is the absolute temperature, and *R* is the universal gas constant (8.314 J/mol·K).

To calculate the kinetic parameters, it is essential to establish the conversion fraction, which is determined as the ratio of the current mass change to the initial mass (3):

3 $$\alpha =\frac{{m}_{i}-{m}_{\alpha }}{{m}_{i}}$$

where *m*_*i*_*, m*_*f,*_ and *m*_*α*_ are the initial, final, and current mass of the sample, respectively.

4 $${m}_{\alpha }-{m}_{f}= {m}_{i}(1-\alpha )-{m}_{f}$$

5 $$r=-\frac{d\left({m}_{\alpha }-{m}_{f}\right)}{dt}={m}_{i}\frac{d\alpha }{dt}=Bexp\left(\frac{-{E}_{A}}{RT}\right)F\left({m}_{i}(1-\alpha )-{m}_{f}\right)$$

The constant heating rate (*β*) may be defined as:

6 $$\beta =\frac{dT}{dt}=constant$$

7 $$\frac{1}{\frac{dT}{d\alpha }}= \frac{B}{\beta {m}_{i}}exp\left(\frac{-{E}_{A}}{RT}\right)F\left({m}_{i}(1-\alpha )-{m}_{f}\right)$$

Substituting:

8 $$A=\frac{B}{{m}_{i}} and f\left(\alpha \right)= F\left({m}_{i}(1-\alpha )-{m}_{f}\right)$$

we obtain:

9 $$\frac{1}{\frac{dT}{d\alpha }}=\frac{A}{\beta }{e}^{\frac{{-E}_{A}}{RT}}f\left(\alpha \right)$$

## Theoretical approach: determination of the form of the function *f*(α)

The description of the thermal degradation process is based on the Arrhenius equation in which the unknown values—activation energy *E*_*A*_, constant *A*, and the model function *f*(α) characterizing the physical process—should be determined on the basis of the temperature function *T* = *T*(α), where α is the conversion fraction. This is the inverse factor problem with three unknowns: *E*_*A*_, *A*, *f*(α); the function *f*(α) is treated here as one of the unknowns. Moreover, the explicit form of the model function *f*(α) is unknown. Thus, the basic problem is to determine the class of functions to which the model function belongs, and then to define an approximation of the function *f*(α) parametrically. Next, the function *f*(α) will be presented in the form of a polynomial of degree *n*.

The Arrhenius equation defines the process of thermal degradation of spongin in the form:

10 $$\frac{1}{\frac{dT}{d\alpha }}=\frac{A}{\beta }{e}^{\frac{{-E}_{A}}{RT}}f\left(\alpha \right){\alpha }\in (\mathrm{0,1})\mathrm{ T}^{\prime}({\alpha })\in \mathrm{C}(\mathrm{0,1})\mathrm{ f}(\mathrm{\alpha })\in \mathrm{C}\propto (\mathrm{0,1})\mathrm{ R},\upbeta ,\mathrm{EA }\in \mathrm{R}$$

Note that after transforming the Eq. (11) by taking logarithms, we obtain it in the linear form in the system ($\frac{1}{T}$, $\mathrm{ln}\frac{\beta \left(\frac{d\alpha }{dT}\right)}{f(\alpha )} )$:

11 $$\mathrm{ln}\frac{\beta \left(\frac{d\alpha }{dT}\right)}{f(\alpha )}=\left(- \frac{{E}_{A}}{R}\right)\frac{1}{T}+lnA$$

**Figure 1:**
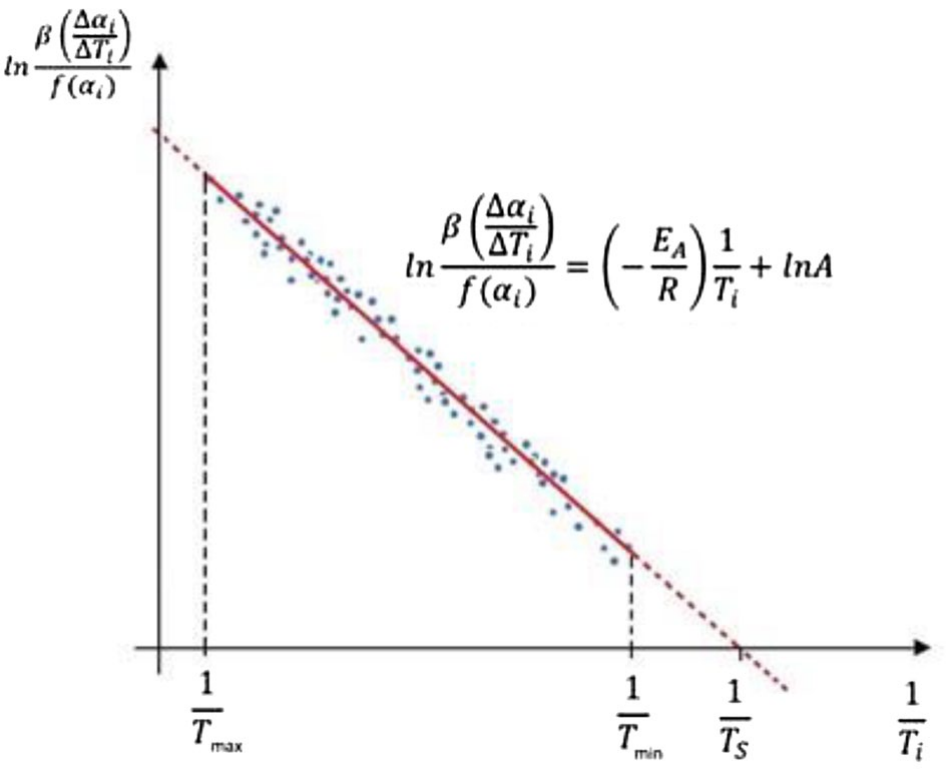
The idea of determining the temperature *T*_*s*_ for calculation of the constant *A*.

The intersection of the line (12) with the axis 1/*T* (Fig. 1) occurs when $\mathrm{ln}\frac{\beta \left(\frac{d\alpha }{dT}\right)}{f(\alpha )}$ = 0, which corresponds to the temperature *T*_*s*_ is 0; therefore

12 $$0= \left(- \frac{{E}_{A}}{R}\right)\frac{1}{\mathrm{ Ts}}+ lnA, \, \, \, {\mathrm{ hence }A = e}^{\frac{{E}_{A}}{R{T}_{s}}}$$

and in further calculations we will use the temperature *T*_*s*_. On the other hand, the expression $\mathrm{ln}\frac{\beta \left(\frac{d\alpha }{dT}\right)}{f(\alpha )}=0$ is equal to zero and hence β = T’(α*)·(α*), where α* corresponds to the unknown temperature *T*_*s*_.

Equation (12) for pairs of experimental data (*T*(α_*i*_), *f*(α_*i*_)), *i* = 1,2,…,*N*_*experiment*_, is graphically presented in Fig. 1. If the experimental data were without errors, then the measurement points would lie on a straight line.

For further considerations, let us transform Eq. (11) by expressing the constant *A* in terms of the temperature *T*_*s*_ based on (13). Then Eq. (11) takes the form

13 $$\frac{\beta }{\frac{dT}{d\alpha }}*{e}^{\frac{-{E}_{A}}{R{T}_{s}}+\frac{{E}_{A}}{RT}}=f\left(\alpha \right), \alpha \in <\mathrm{0,1}>$$

which leads to the conclusion that the model function *f*(α) is exponential, and after taking logarithms we obtain

14 $$\frac{ln\frac{\beta }{f(\alpha ){T}^{^{\prime}}}}{\frac{{E}_{A}}{R{T}_{s}}-\frac{{E}_{A}}{RT}}=1$$

which is the quality criterion for selecting the model function *f*(α).

For the measurement data (*T*(α_*i*_), *f*(α_*i*_)), *i* = 1,2,…,*N*_*experiment*_, and the adopted form of the function *f*(α), the relationship (14) will be subject to some error δ_*i*_:

15 $${\updelta }_{i}=\frac{\beta }{\frac{dT}{d\alpha }}\cdot {e}^{\frac{-{E}_{A}}{R{T}_{s}}+\frac{{E}_{A}}{RT}}-f\left(\alpha \right),{\alpha }={{\alpha }}_{i}$$

Equation (16) is the basis for the definition of the method of determining the unknowns *E*_*A*_, *A*, *f*(α).

Since the right-hand side of Eq. (14) is an exponential function, the next logical step involves the representation of *f*(α) as an exponential function. Taking the logarithm of Eq. (14), we obtain

16 $$lnf\left(\alpha \right)=\mathrm{ln}(\frac{\beta }{\frac{\mathit{dT}}{\mathit{d\alpha }}}\cdot {e}^{-\frac{{E}_{A}}{R{T}_{s}}+\frac{{E}_{A}}{\mathit{RT}}})={a}_{0}+{a}_{1}\alpha +{a}_{2}{\alpha }^{2}+\dots +{a}_{n}{\alpha }^{n} $$

The quotient $\frac{\beta }{\frac{dT}{d\alpha }}$ can also be presented as an exponential function, so $\frac{\beta }{\frac{dT}{d\alpha }}={e}^{ln\frac{\beta }{{T}^{^{\prime}}}}$, where ${T}^{^{\prime}}=\frac{dT}{d\alpha }$ , thus

17 $$lnf\left(\alpha \right)=\mathrm{ln}\frac{\beta }{T{^{\prime}}}-\frac{{E}_{A}}{R{T}_{s}}+\frac{{E}_{A}}{\mathit{RT}}={a}_{0}+{a}_{1}\alpha +{a}_{2}{\alpha }^{2}+\dots +{a}_{n}{\alpha }^{n}$$

18 $$f\left(\alpha \right)={e}^{{a}_{0}+{a}_{1}\alpha +{a}_{2}{\alpha }^{2}+\dots +{a}_{n}{\alpha }^{n}}$$

The parameters ${a}_{0}, {a}_{1},\dots , {a}_{n},{E}_{A, }{T}_{s}$ are unknown; the number of these parameters is $n+3$. Assuming the continuity of the function *T*(α), the integral fit function takes the form

19 $$I({a}_{0}, {a}_{1},\dots , {a}_{n},{E}_{A, }{T}_{s})={\int }_{{\alpha }_{0}}^{{\alpha }_{k}}{\left[{e}^{{a}_{0}+{a}_{1}\alpha +{a}_{2}{\alpha }^{2}+\dots +{a}_{n}{\alpha }^{n}}-{e}^{\mathrm{ln}\frac{\beta }{T{^{\prime}}(\alpha )}-\frac{{E}_{A}}{R{T}_{s}}+\frac{{E}_{A}}{RT\left(\alpha \right)}}\right]}^{2}d\alpha =min$$

However, during the thermal degradation process, we obtain a set of measurement points (α_*i*_, *T*_*i*_), *i* = 1,2,…, *N*, where *N* is the number of measurement points, *N* >> *n*. Thus, an overdetermined system of equations determines the sought parameters. The above integral for the discrete distribution can be expressed, for example, by integration by the method of rectangles, which leads to the minimization of the functional:

20 $$I({a}_{0}, {a}_{1},\dots , {a}_{n},{E}_{A, }{T}_{s})=\sum_{i=1}^{N}{\left({e}^{{a}_{0}+{a}_{1}{\alpha }_{i}+{a}_{2}{{\alpha }_{i}}^{2}+\dots +{a}_{n}{{\alpha }_{i}}^{n}}-{e}^{\mathrm{ln}\frac{\beta }{T{^{\prime}}(\alpha )}-\frac{{E}_{A}}{R{T}_{s}}+\frac{{E}_{A}}{RT\left({\alpha }_{i}\right)}}\right)}^{2}\cdot \left({\alpha }_{i}-{\alpha }_{i-1}\right)=min$$

where *N* = *N*_*experiment*_.

The various methods of integrating the functional (20) on the results obtained for the kinetic parameters are shown in Supplementary information, Tables 2 and 3. As presented, the primary issue is to determine an approximation of the function *f*(α), that is, to determine whether to approximate using a polynomial equation:

- The exponential form of the function *f*(α) according to Eq. (10), further Eq. (18).
- The function *f*(α) as represented by Eq. (17).

### Determination of the activation energy *E*_*A*_ and temperature *T*_*S*_

#### Minimization of the fit functional (19)

The matching functional (19) can be written as follows:

21 $$I\left({E}_{A, }{T}_{s}, {a}_{0}, {a}_{1},\dots , {a}_{n}\right)=\sum_{i=1}^{N}{\left({e}^{{a}_{0}+{a}_{1}{\alpha }_{i}+{a}_{2}{{\alpha }_{i}}^{2}+\dots +{a}_{n}{{\alpha }_{i}}^{n}}-{e}^{\mathrm{ln}\left(\frac{\beta }{{T}^{^{\prime}}\left({\alpha }_{i}\right)}\right)-\frac{{E}_{A}}{R{T}_{s}}+\frac{{E}_{A}}{RT\left({\alpha }_{i}\right)}}\right)}^{2}\cdot \left({\alpha }_{i}-{\alpha }_{i-1}\right)=min$$

or in a more compact form:

22 $$I=\sum_{i=1}^{N}{\left({e}^{{a}_{0}+{a}_{1}{\alpha }_{i}+{a}_{2}{{\alpha }_{i}}^{2}+\dots +{a}_{n}{{\alpha }_{i}}^{n}}-{e}^{{g}_{i}}\right)}^{2}\cdot \left({\alpha }_{i}-{\alpha }_{i-1}\right)=\mathrm{min}, N\ge n, {g}_{i}=\mathrm{ln}\left({\frac{\beta }{{T}^{^{\prime}}}\left({\alpha }_{i}\right)}\right)-\frac{{E}_{A}}{R{T}_{s}}+\frac{{E}_{A}}{RT\left({\alpha }_{i}\right)}$$

The approach described in^48^ was used to minimize the functionals (23). This method requires the user to define the starting values of the sought variables ${a}_{0},{a}_{2} \ldots ,{a}_{n}$, *E*_*A*_, *T*_*S*_.

#### Minimizing the fit functional (19)

Let us check the quality of the fit of the polynomial $ln {f\left({\alpha }_{i}\right)=a}_{0}+{a}_{1}{\alpha }_{i}+{a}_{2}{\alpha }_{i}^{2}+\dots +{a}_{n}{\alpha }_{i}^{n} for i=\mathrm{1,2},.$.,*N*, minimizing the mean-square function (19) for the sequence of values *T*(${\alpha }_{i}$), β, *E*_*A*_ and *T*_*S*_, measured for spongin-based skeletons. The function *T* = *T*(α) is plotted in Fig. S2, and its first derivative T’ = d*T*/dα in Fig. S1. The details regarding the experimental measurements are given in Supplementary materials.

The results of the functional optimization are shown in Fig. S1A. The differences between the curves result from temperature measurement errors in *T* = *T*(α) and the numerical calculation of its derivative d*T*/dα, as well as the constraints imposed on the variables in the optimization process. Figure S1B shows the graph of the functional ln(β/(*f*(α) · *T’*_experiment) depending on the variable 1/*T*. The correct selection of the function *f*(α) (as an exponential function) ensured the linearity of the graphs shown.

Figures S1–S3 correspond to the measuring interval α_0_ = 0.0867; α_end_ = 0.742. Above the value α = 0.6 the first derivative of the temperature function *T* = *T*(α) begins to undulate, which is inconsistent with the physical process. Therefore, this value was used as α_end_. It should be noted that the measuring interval was chosen to correspond to the chemical process of thermal degradation. For values of α lower than 0.0867 the physical process of water evaporation is taking place, and therefore that part was excluded from evaluation. Figures S4–S6 show the same graphs as in Figs. S1–S3, but with the values α_0_ = 0.0867 and α_end_ = 0.6. There is no ripple of the first derivative of temperature in this range.

Based on the above considerations, the characteristic values of the thermal degradation process are listed below in numerical form in Table 1.

**Table 1:** Numerical values of kinetic parameters determined by the new approach.

	β (°C/min)	*E*_*A*_ (J/mol K)	*T*_*S*_ (K)	*A*	α_0_	α_end_
Degree of polynomial: *N*_*polynom*_ = 6	2.5	50.593	246.48	5.26 × 10^10^	0.0867	0.742
	5.0	53.903	255.35	1.06 × 10^11^	0.0816	0.68
	10.0	50.246	248.00	3.82 × 10^10^	0.0802	0.693
	20.0	50.989	251.47	3.89 × 10^10^	0.0958	0.699
Degree of polynomial: *N*_*polynom*_ = 7	2.5	51.115	247.70	6.00 × 10^10^	0.0867	0.742
	5.0	53.165	253.74	8.78 × 10^10^	0.0816	0.680
	10.0	49.401	246.02	3.08 × 10^10^	0.0802	0.693
	20.0	52.197	254.09	5.36 × 10^10^	0.0958	0.699

**Figure 2:**
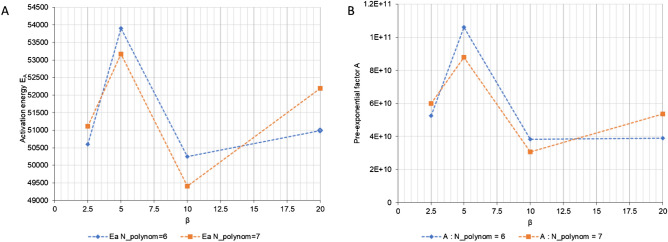
Influence of the degree of the polynomial equation on the graphs of activation energy (**A**) and the pre-exponential coefficient (**B**) as functions of heating rate.

The above values are presented graphically in Fig. 2. Comparison of the numerical values resulting from application of sixth-degree (*N*_*polynom*_ = 6) and seventh-degree (*N*_*polynom*_ = 7) polynomial equations reveals insignificant differences in the determined activation energy values. The constant *A* can be determined using the relationship *A* = ${e}^{\frac{{E}_{A}}{R{T}_{s}}}$; its value is influenced by errors in determining the activation energy and the temperature *T*_*S*_.

**Figure 3:**
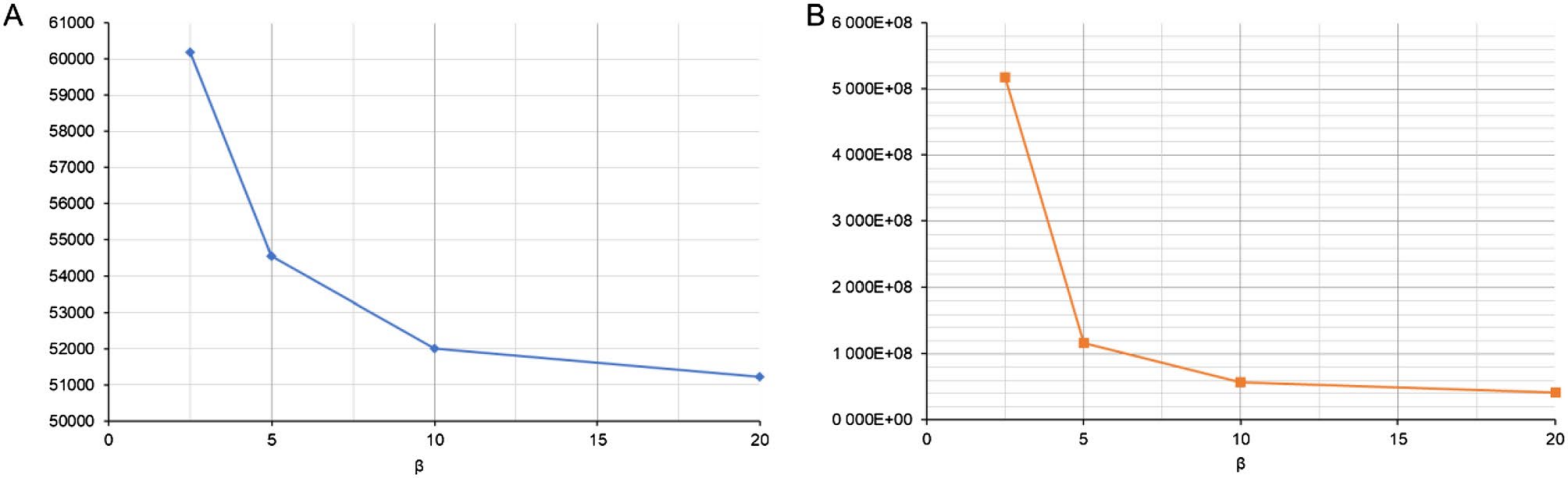
Graphs of activation energy (**A**) and the pre-exponential factor (**B**) as functions of the rate of thermal degradation for the measured data.

The graphs shown in Fig. 2 correspond to different values of the beginning (α_0_) and end (α_end_) conversion degree of the thermal degradation process. For the same values of the interval < α_0_, α_end_ > but different values of the rate of the degradation process, the values of the activation energy *E*_*A*_ and the pre-exponential constant *A* for the measurement data are presented in Fig. 3.

### Comparison of classical model functions with a polynomial function reflecting the real process of thermal degradation

Let us denote the model function as *f*_*model*_(α) and the polynomial by *f*(α). Figure S9 shows graphs of the Zhuravlev model function and the polynomial function for the thermal degradation of spongin-based scaffolds using a heating rate equal to 2.5. The closeness of these functions can be expressed by a functional (24):

23 $$I\left(\gamma \right)=\sum_{i}{\left[{f}_{model}\left({\alpha }_{i}\right)-\gamma f({\alpha }_{i})\right]}^{2}min\to \frac{1}{2}\frac{dI}{d\gamma }=\sum_{i}f\left({\alpha }_{i}\right){\left[{f}_{model}\left({\alpha }_{i}\right)-\gamma f\left({\alpha }_{i}\right)\right] }=0$$

hence

24 $$\gamma =\frac{\sum_{i}{f({\alpha }_{i})f}_{model}\left({\alpha }_{i}\right)}{\sum_{i}{f}^{2}({\alpha }_{i})}$$

**Figure 4:**
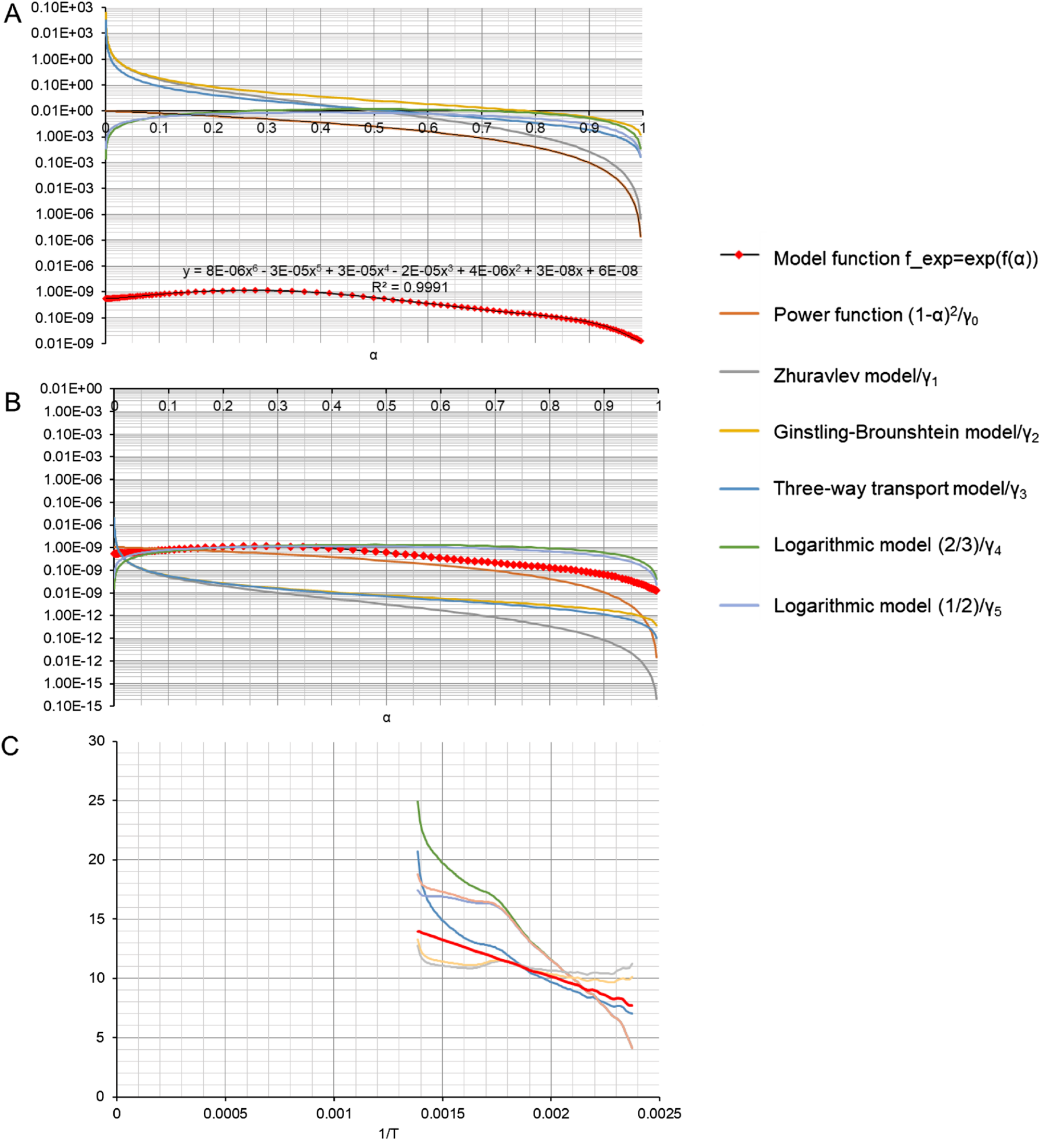
Comparison of the evaluated model *f*_*exp*_ = exp(*f*(α)) with theoretical models on a semi-logarithmic scale for β = 2.5 (**A**); comparison of the evaluated function exp(*f*(α)) with other model functions, with the scaling factor || γ · *f*(α)-*f*_*model*_(α)||^2^ = min(γ) for β = 2.5, where γ_0_ = 0.94191 × 10^7^, γ_1_ = 0.32135∙10^10^, γ_2_ = 0.32440∙10^10^, γ_3_ = 0.16198∙10^10^, γ_4_ = 0.86725 × 10^7^, γ_5_ = 0.73319 × 10^7^ (**B**); and graphs of ln[β(*d*α/*dT*)/(*f*(α))]—the left-hand side of Eq. (12)—with the following functions substituted for *f*(α): the polynomial function evaluated in this study, and standard model functions multiplied by the scaling factor γ, for β = 2.5.

Thus, when the function *f*(α) is multiplied by γ (or ${f}_{model}\left(\alpha \right)$ is divided by γ), these functions are maximally close to each other in the mean-square sense. Consequently, each model function ${f}_{model}\left(\alpha \right)$ has a corresponding value of the scaling factor γ (Fig. 4).

When the model functions shown in Fig. 4 are used in the Arrhenius equation, they should be multiplied by a scaling factor γ, the value of which is unknown. Thus, the Arrhenius Eq. (11) takes the form:

25 $$\frac{1}{\frac{dT}{d\alpha }}=\frac{A}{\beta }{e}^{\frac{{-E}_{A}}{RT}}{f}_{model}\left(\alpha \right)\cdot \gamma $$

Thus, for the assumed model function *f*_*model*_(α), the unknown quantities in the Arrhenius equation are the activation energy *E*_*A*_, the constant γ, and the pre-exponential factor *A*.

The pre-exponential factor *A* is determined using the relationship *A* = ${e}^{\frac{{E}_{A}}{R{T}_{s}}}$; its value is influenced by errors in determining the activation energy and the temperature *T*_*S*_.

### Influence of random errors of measurement of *T* = *T*(α) on the derivative ${{{T}}}^{^{\prime}}({{\upalpha}})=\frac{{{d}}{{T}}}{{{d}}{\alpha }}$

During the thermal degradation process, the derivative ${T}^{^{\prime}}$ is a positive function; therefore, for an undisturbed (exact) temperature graph *T* = *T*(α), the slope of the tangent line is less than 90°, thus tan(*T'*(α)) > 0—as shown in Fig. S11. Disturbance of *T*(α) by a random error (disturbed measurement points are marked in Fig. S11) may cause a situation where tan(*T'*(α)) < 0, which does not correspond to the physics of the thermal degradation phenomenon. Thus, the question arises: how to determine the set of permissible random errors of temperature so that the disturbed distribution meets the condition $T^{\prime}({\upalpha })>0$?

### The inverse problem of determining the permissible set of temperature disturbances based on disturbance of its derivative while maintaining the physical condition ${{T}}^{\prime}({{\upalpha}})>0$

The need to solve the above problem arose from the analysis of the temperature graph *T* = *T*(α) (Fig. S11), where the inclination of the secant passing through the measured temperature points (randomly generated) is shown.

Large measurement errors may cause *T'*(α) to take negative values, because tan(*T'*(α)) < 0, despite the non-negative nature of the function *T'*(α) resulting from the physical process. Therefore, the question arises: which errors of disturbance of the derivative *T'*(α), fulfilling the condition that it must be non-negative, correspond to errors of temperature disturbance? The following identity will be used to solve this problem:

26 $$T\left(\alpha \right)=T\left({\alpha }_{0}\right)+{\int }_{{\alpha }_{0}}^{\alpha }\frac{dT(\alpha )}{d\alpha }d\alpha $$

For the disturbed course of the derivative ${\left(\frac{dT(\alpha )}{d\alpha }\right)}_{disturbed}$ the derivative error has the form

27 $${\left(\frac{dT(\alpha )}{d\alpha }\right)}_{disturbed}=\frac{dT(\alpha )}{d\alpha }+\delta \left(\frac{dT\left(\alpha \right)}{d\alpha }\right)=\frac{dT\left(\alpha \right)}{d\alpha }\cdot \left(1+\omega \cdot p\left(\alpha \right)\right)d\alpha $$

Thus

28 $$T\left(\alpha \right)+\delta T\left(\alpha \right)=T\left({\alpha }_{0}\right)+{\int }_{{\alpha }_{0}}^{\alpha }{\left(\frac{dT(\alpha )}{d\alpha }\right)}_{disturbed}d\alpha =T\left({\alpha }_{0}\right)+{\int }_{{\alpha }_{0}}^{\alpha }\frac{dT(\alpha )}{d\alpha }\left(1+\omega \cdot p\left(\alpha \right)\right)d\alpha $$

where *p*(α) is a random variable $p\left(\alpha \right)\epsilon <-\mathrm{1,1}>$, while ω $\epsilon <\mathrm{0,1}>$ denotes the margin of error.

Because $1+\omega \cdot p\left(\alpha \right)>0$, for $\frac{dT(\alpha )}{d\alpha }>0$ (physical process), the integral of the expression (14) is always positive, and finally, the random distribution of temperature measurement error after subtracting the identity (27) from Eq. (29) is:

29 $$\delta T\left(\alpha \right)= \omega {\int }_{{\alpha }_{0}}^{\alpha }\frac{dT\left(\alpha \right)}{d\alpha }p(\alpha )d\alpha $$

On disturbing the temperature with the error (27) the derivative is always $\frac{dT}{d\alpha }>0$, which is consistent with the physical phenomenon.

The results of calculations of the activation energy *E*_*A*_ and the pre-exponential factor *A* for disturbed and undisturbed data described by the parameter δ_random_T’ will be used in the process of minimizing the mean square integral (20) for various approximations.

The following notations will be used:

- ls_exp_parabolic—integration using the parabolic arches method of the function
  30 $${h\left(\alpha \right)=\left[{e}^{{a}_{0}+{a}_{1}\alpha +{a}_{2}{\alpha }^{2}+\dots +{a}_{n}{\alpha }^{2}}-{e}^{\mathrm{ln}\frac{\beta }{{T\left(\alpha \right)}^{^{\prime}}}-\frac{{E}_{A}}{R{T}_{s}}+\frac{{E}_{A}}{RT\left(\alpha \right)}}\right]}^{2}$$
- ls_exp_trapez—integration using the trapezoidal method of the function *h*(α).
- ls_exp_rectangular—integration using the rectangle method of the function *h*(α).
- ls_polynom_parabolic—integration by the parabolic arches method of functions from the difference of exponents
  $${{g\left(\alpha \right)=[a}_{0}+{a}_{1}\alpha +{a}_{2}{\alpha }^{2}+\dots +{a}_{n}{\alpha }^{n}-\mathrm{ln}\frac{\beta }{T^{{\prime}}(\alpha )}-\frac{{E}_{A}}{R{T}_{s}}+\frac{{E}_{A}}{\mathit{RT}(\alpha )}]}^{2}$$
- ls_polynom_trapez—integration by the trapezoidal method of functions from the difference of exponents *g*(α).
- ls_polynom_rectangular—integration by the rectangle method of functions from the difference of exponents *g*(α).
- ls_algebraic—the sum of squares from the difference of exponents at successive measurement points ${{[a}_{0}+{a}_{1}{\alpha }_{i}+{a}_{2}{{\alpha }_{i}}^{2}+\dots +{a}_{n}{{\alpha }_{i}}^{n}-\mathrm{ln}\frac{\beta }{{T}^{{\prime}}\left({\alpha }_{i}\right)}-\frac{{E}_{A}}{R{T}_{s}}+\frac{{E}_{A}}{\mathit{RT}\left({\alpha }_{i}\right)}]}^{2}, i=1,{N}_{experiment}$

The results obtained with the use of the various integration methods for undisturbed and disturbed data are presented in Tables 2 and 3.

**Table 2:** Numerical values of kinetic parameters determined using various integration methods for β = 20, δ_random_T’ = 0.0, *N*_*polynom*_ = 7.

Numerical integration	*A*	*T*_*S*_ (K)	*E*_*A*_ (J/mol K)
ls_exp_parabolic	0.53621 × 10^11^	254.09	52.197
ls_exp_trapez	0.53237 × 10^11^	254.03	52.169
ls_exp_rectagular	0.49339 × 10^11^	253.28	51.855
ls_polynom_parabolic	0.22700 × 10^10^	293.35	52.549
ls_polynom_trapez	0.20292 × 10^10^	265.52	52.523
ls_polynom_rectangular	0.16769 × 10^11^	295.68	51.977
ls_algebraic	0.30799 × 10^9^	278.41	48.055

**Table 3:** Numerical values of kinetic parameters determined using various integration methods for β = 20, δ_random_T’ = 0.1, *N*_*polynom*_ = 7.

Numerical integration	*A*	*T*_*S*_ (K)	*E*_*A*_ (J/mol K)
ls_exp_parabolic	0.14151 × 10^12^	262.46	56.033
ls_exp_trapez	0.13391 × 10^12^	261.96	55.805
ls_exp_rectagular	0.13343 × 10^12^	261.86	55.776
ls_polynom_parabolic	0.95852 × 10^9^	302.00	51.932
ls_polynom_trapez	0.96869 × 10^9^	309.21	53.199
ls_polynom_rectangular	0.25254 × 10^11^	263.34	52.447
ls_algebraic	0.84421 × 10^9^	278.41	47.581

**Figure 5:**
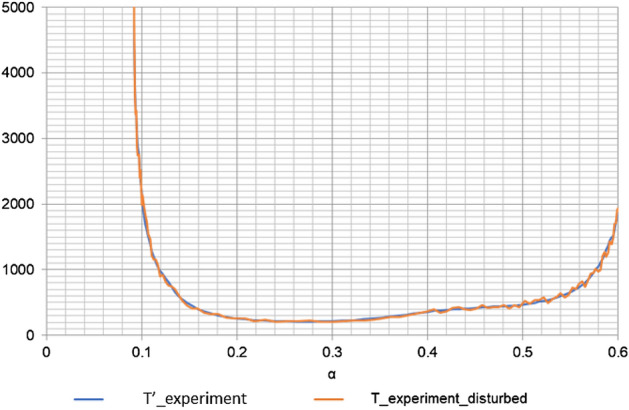
Quasi-exact graph of the temperature derivative and with 10% random disturbance for β = 2.5.

Figure 5 explains why the result of ls_algebraic numerical integration for the activation energy *E*_*A*_ differs from the others. The reason is the constant value of the length of the integration interval: *α(i) – α(i – 1)* = *const*. In all other integration methods, the length of the integration interval is variable, as shown in Fig. 5.

## Conclusions

In this study, a mathematical description of the thermal degradation of spongin-based scaffolds has been given. In the investigation, the model function *f*(α) was properly selected, considering the experimental data, and consequently, the development of a family of theoretical models was possible. Moreover, it was proved that the classical solid-state models do not lead to an exact linear correlation when they are used to describe the experimental function *f*(α). Likewise, in the course of the study, a better method of minimizing the functional has been developed, so that the constant *A* decreases monotonically with an increase in α. Consequently, the polynomial model evaluated in this work was compared with known theoretical models. Finally, the numerical values of activation energy and pre-exponential factors were obtained using approximations of the function *f*(α) with 6th- and 7th-degree polynomials. Interestingly, despite the different approximations used, the differences in the results were not significant. Moreover, the use of mean square minimization made it possible to solve the inverse problem for the Arrhenius equation with unknown transformation function *f*(α) and pre-exponential constant *A*. In addition, this work examines the sensitivity of the solution to the inverse problem to errors in temperature measurement, within the class of disturbances corresponding to physically possible values of the temperature derivative *T'*(α).

This study provides deep insight into the mathematical description of the thermal degradation process. On the basis of the classical approach and currently used temperature integral approximations, a new pathway of investigation has been indicated. However, further investigation is essential to develop new, meaningful and reliable methods of kinetic analysis for thermal reactions, which should be related to accurate measurements in which values of the parameter α depend directly on the rate of thermal reaction. Therefore, the proper application of chemical principles to describe the mechanisms of chemical reaction and identify the controlling factors is required. It is necessary to find a “compromise” between the mathematical approach and chemical principles to properly describe the chemical processes occurring during thermal degradation, because mathematical methods are not sufficient to investigate the chemical changes taking place during thermal degradation. Additionally, relevant information together with kinetic evidence would lead to more reliable conclusions.

Likewise, in the current calculation methods the interpretation of the kinetics is based on solid-state reaction models; therefore, such models might not describe precisely the complicated mechanistic behaviour of some materials of biological origin. The pyrolysis reaction may be controlled by a very complex mechanism or even by a hitherto unknown one. The implementation of a new approach to comparative or relative kinetic analysis opens up new possibilities and enables more detailed evaluation of this problem. Consequently, the method presented in this work may serve as inspiration for further work regarding the mathematical description of thermal degradation processes for other types of biomass.

## Supplementary Information

Supplementary Figures.

## Data Availability

All data generated or analyzed during this study are included in this published article [and its supplementary information file].

## Abbreviations

*E*_*A*_: Activation energy
*A*: Pre-exponential factor
*f*(α): Model function characterizing the process
*T*: Temperature
*T*_*S*_: Characteristic temperature
*R*: Gas constant
*a*_*n*_: Unknown parameter
*N*: Number of measurement points
*N*_*polynom*_: Degree of polynomial
$I({a}_{0}, {a}_{1},\dots , {a}_{n},{E}_{A, }{T}_{s})$: Integral fit function
*T*(α): Function of degree of conversion vs. temperature
*T'*(α): Derivative of the *T*(α) function
*p*(α): A random variable
α: Degree of conversion
$$\beta $$: Heating rate
$$\gamma $$: Scaling factor
$$\delta $$: Random disturbance
$\omega$: Margin of error
