# Supplementary material for: Investigation of the inverse problem for the Arrhenius equation using the example of thermal degradation of spongin-based scaffolds
Source: Sci Rep. 2023 Feb 14;13:2636. doi: 10.1038/s41598-023-29565-1 (PMC9929043; doi:10.1038/s41598-023-29565-1)
Supplement: Supplementary file 1 — Supplementary Figures. [file 41598_2023_29565_MOESM1_ESM.docx]

Supplementary Materials

**Investigation of the inverse problem for the Arrhenius equation using the example of thermal degradation of spongin-based scaffolds**

Sonia Żółtowska^1^, Michał Ciałkowski^2^, Krzysztof Alejski^1^, Teofil Jesionowski^1,*^

^1^ Poznan University of Technology, Faculty of Chemical Technology, Institute of Chemical Technology and Engineering, Berdychowo 4, PL-60965 Poznan, Poland

^2^ Poznan University of Technology, Faculty of Environmental Engineering and Energy, Institute of Thermal Engineering, Piotrowo 3, PL-60965 Poznan, Poland

*Corresponding author: Teofil.jesionowski@put.poznan.pl*


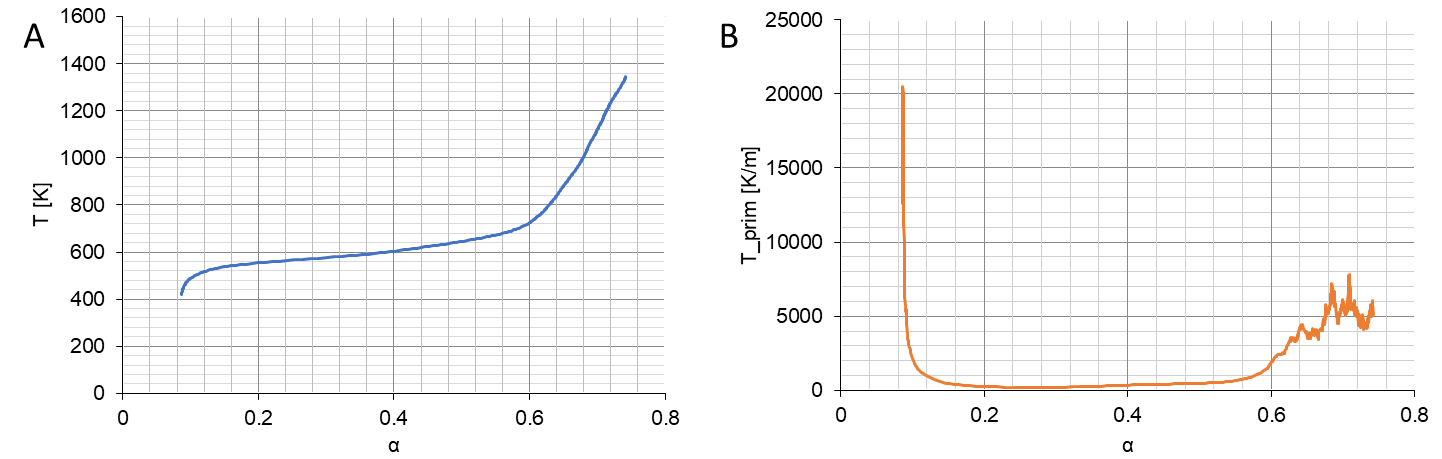


**Fig. S1.** Temperature curve (A) and temperature derivative (B) in the range α_0_=0.0867; α_end_=0.742 for β=2.5.


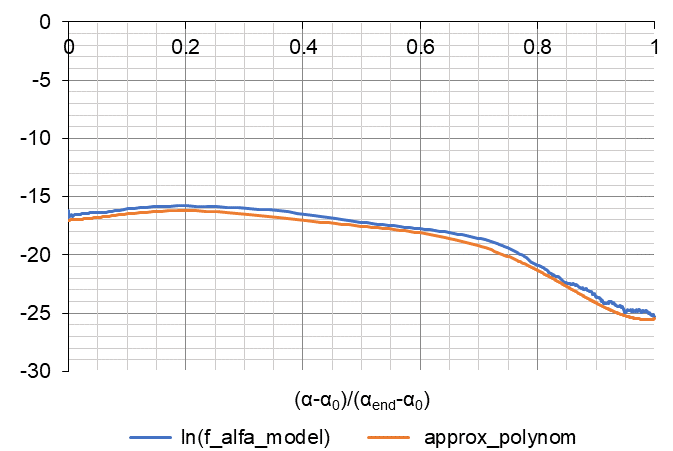


ln(α)_model

**Fig. S2.** Comparison of the approximation of a function (its exponent) ln(*f_model_*(α)) with its polynomial approximation: *N_polynom_*=6; *N*=371; $\ln\left( f\left( \alpha\right)_{model} \right)=\ln(\frac{\beta}{T_{prim}})-(\frac{E_{A}}{R})\cdot\left( \frac{1}{T_{S}}-+\frac{1}{T} \right),$*E_A_*=51028, *T_S_*=270.48, *A*=0.714∙10^10^, α_0_=0.0867, α_end_=0.742.


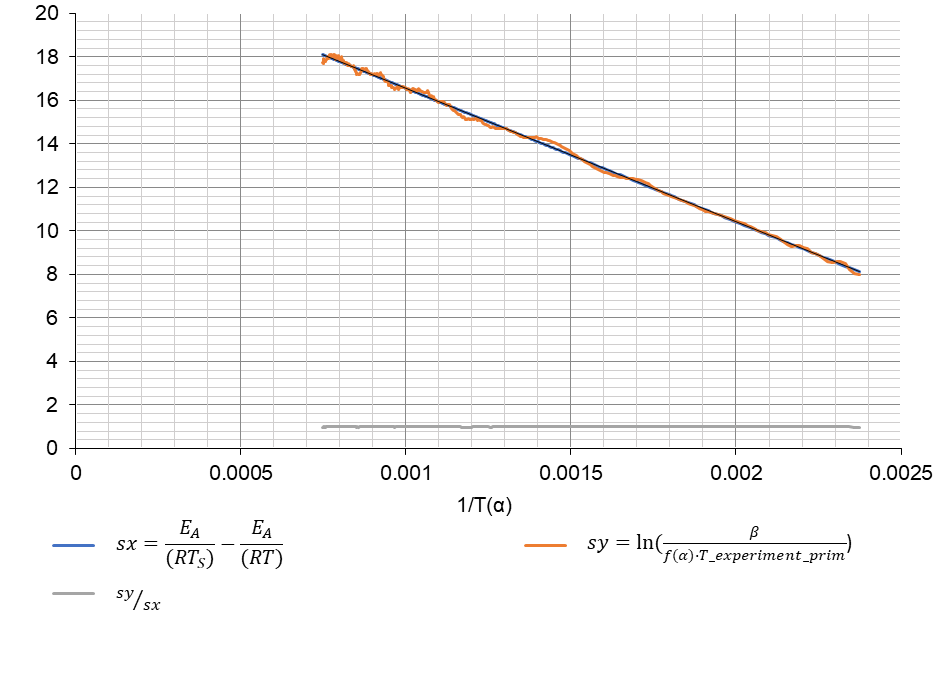


**Fig. S3.** Investigation of the quality of the model function f(α) satisfying the Arrhenius equation (15) for the known values of E_A_=51028, T_S_=270.48 and A=0.714∙1010.


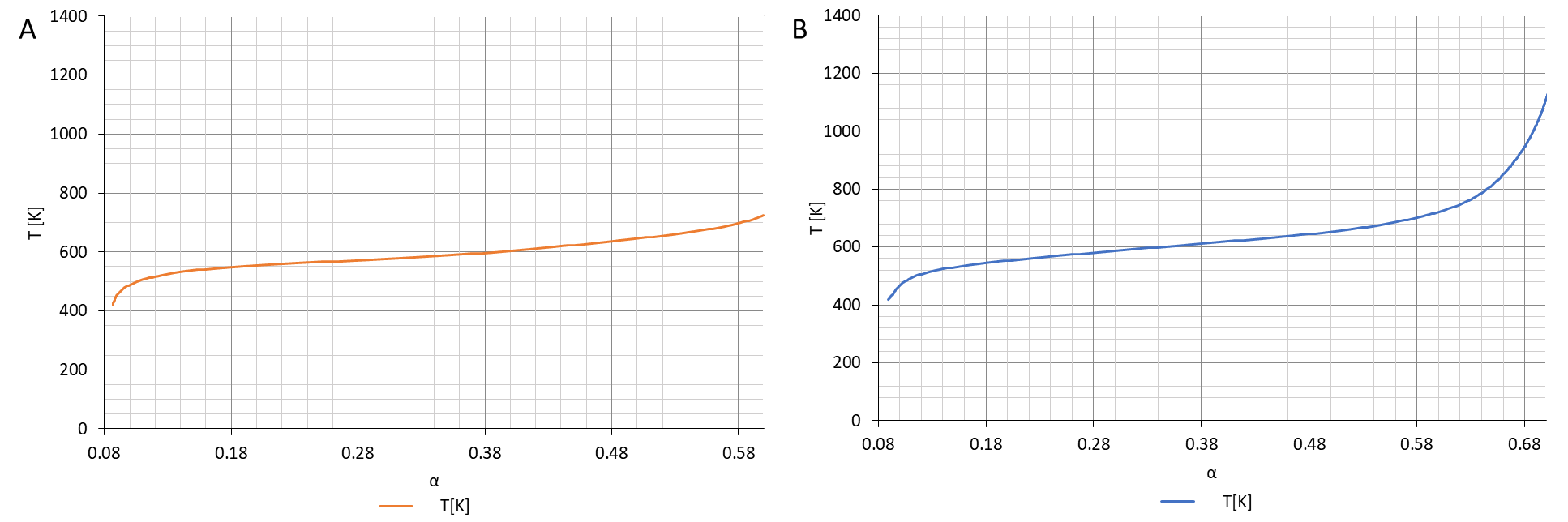


**Fig. S4.** Temperature plots in the range α_0_=0.0867; α_end_=0.6 for β=2.5 (A) and α_0_=0.0958; α_end_=0.699 for β=20 (B).


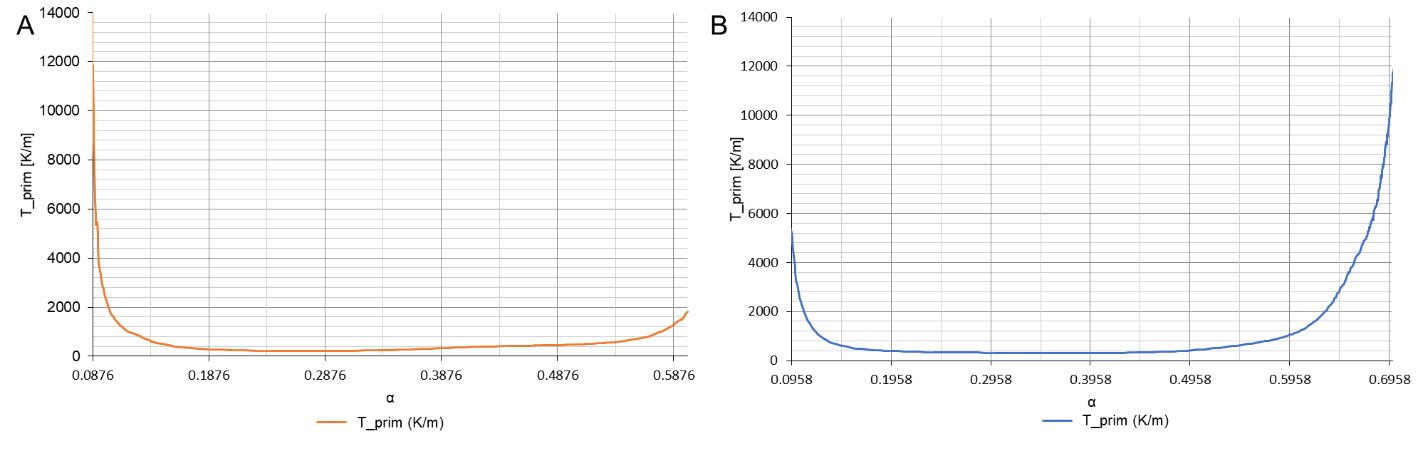


**Fig. S5.** Plots of the first derivative in the range α_0_=0.0867; α_end_=0.6 for β=2.5 (A) and α_0_=0.0958; α_end_=0.699 for β=20 (B).


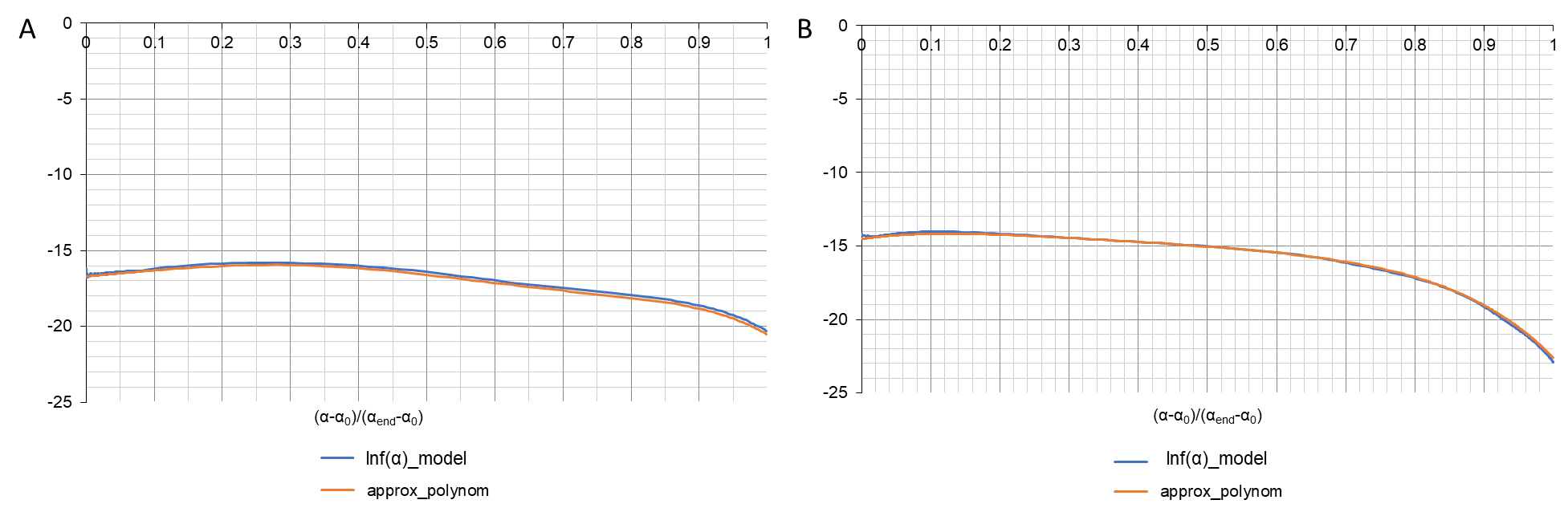


**Fig. S6.** Comparison of the function approximation (exponent) ln(*f_model_*(α)) with its polynomial approximation: *N_polynom_*=6; β=2.5 (A) and β=20 (B).


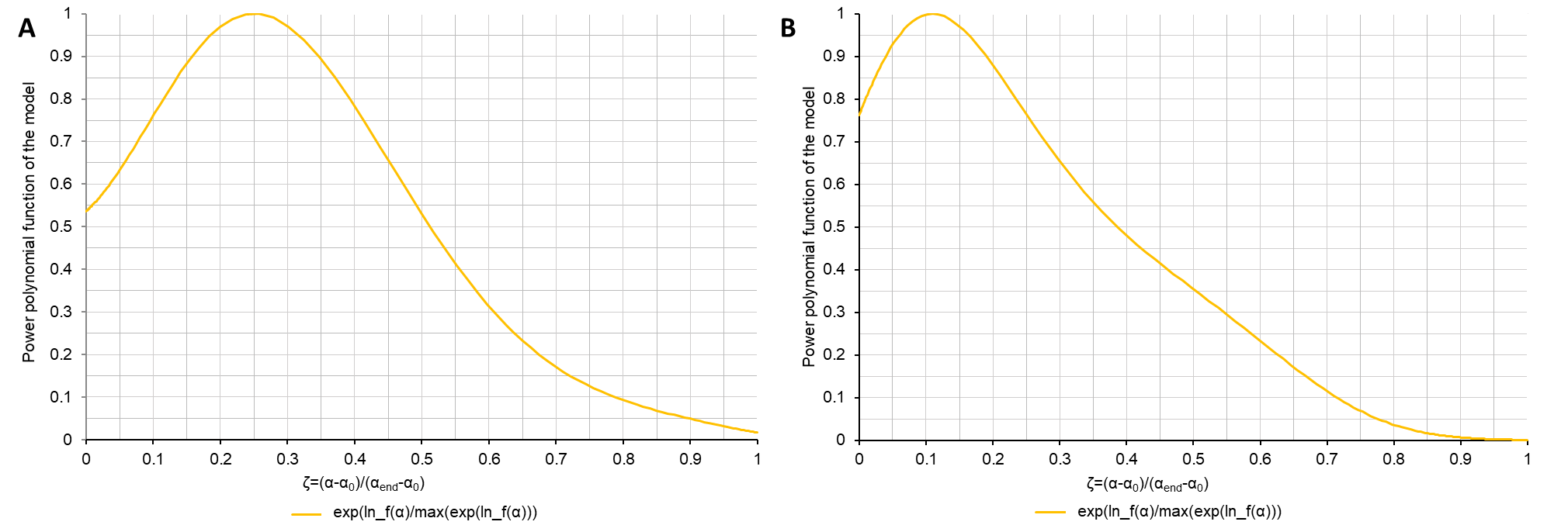


**Fig. S7.** Plots of the relative model function (relative to the maximum value) in the range <α_0_, α_end_> for β=2.5 (A) and β=20 (B).


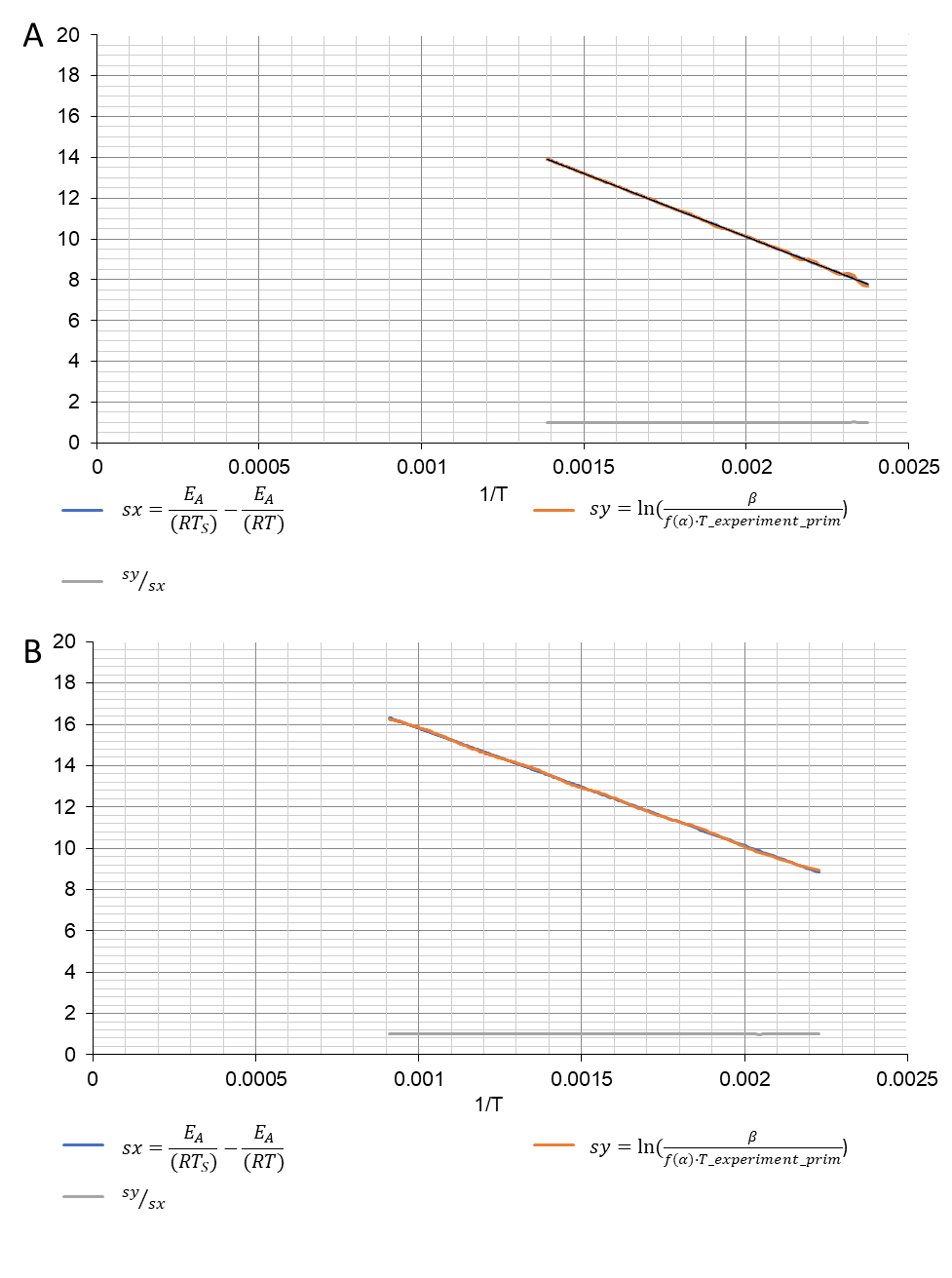


**Fig. S8.** Testing the quality of the model function *f*(α) satisfying the Arrhenius equation (15) for β=2.5 (A) and β=20 (B).


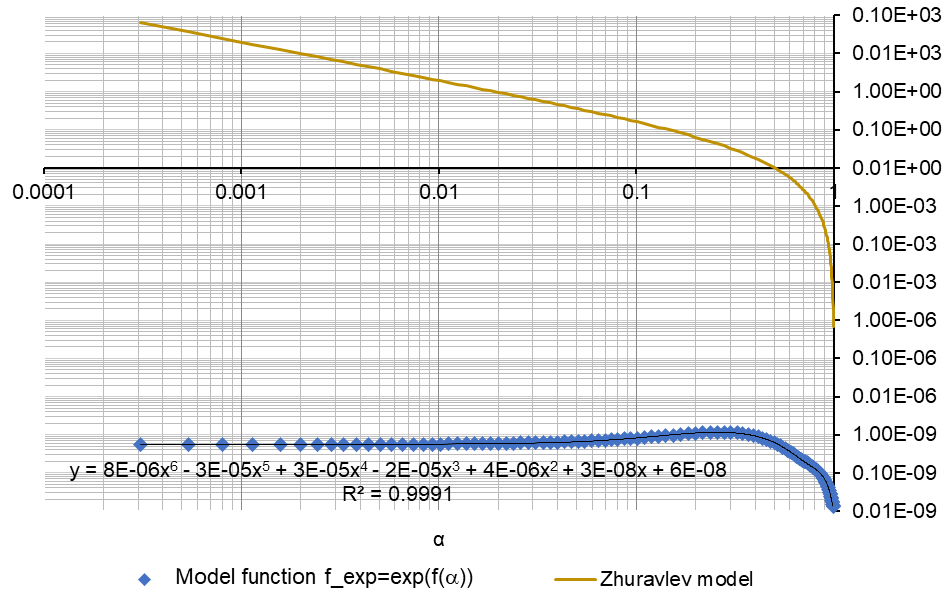


**Fig. S9.** Comparison of the evaluated model *f_exp_*=exp(*f*(α)) with the Zhuravlev function for β=2.5.


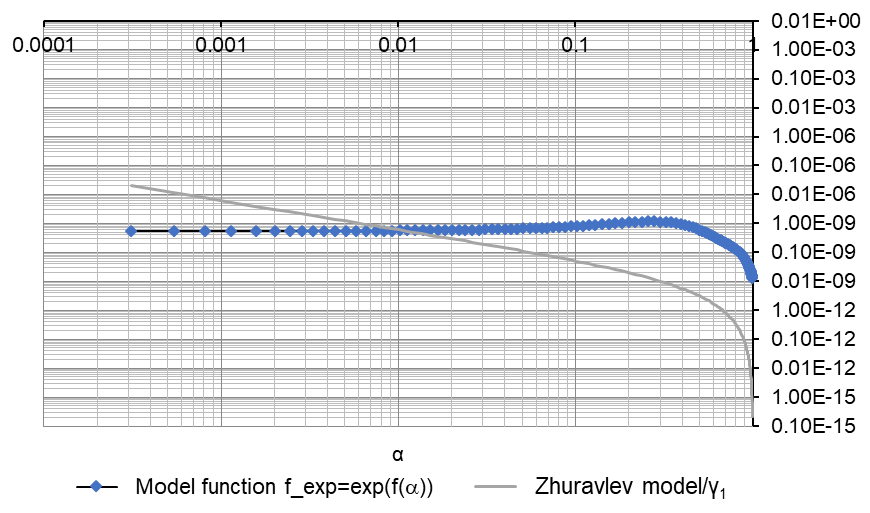


**Fig. S10.** Comparison of the evaluated function exp(*f*(α)) with the scaled Zhuravlev function with the multiplier γ_1_ , so that ||γ_1_$\cdot$*f*(α)-*f_model_*(α)||^2^=min(γ_1_), for β=2.5 and γ_1_= 0.32135∙10^10^.


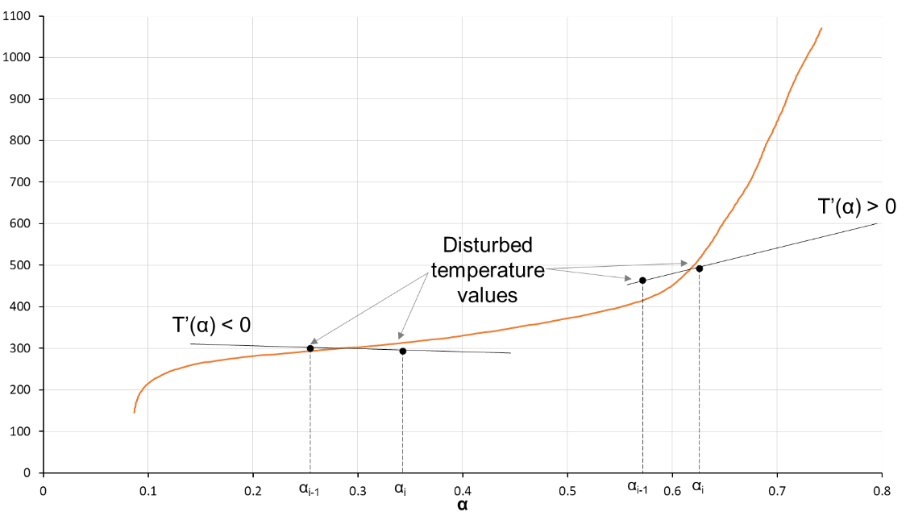


**Fig. S11.** Analysis of the influence of temperature disturbance on the sign of the derivative *T'* (α).

**
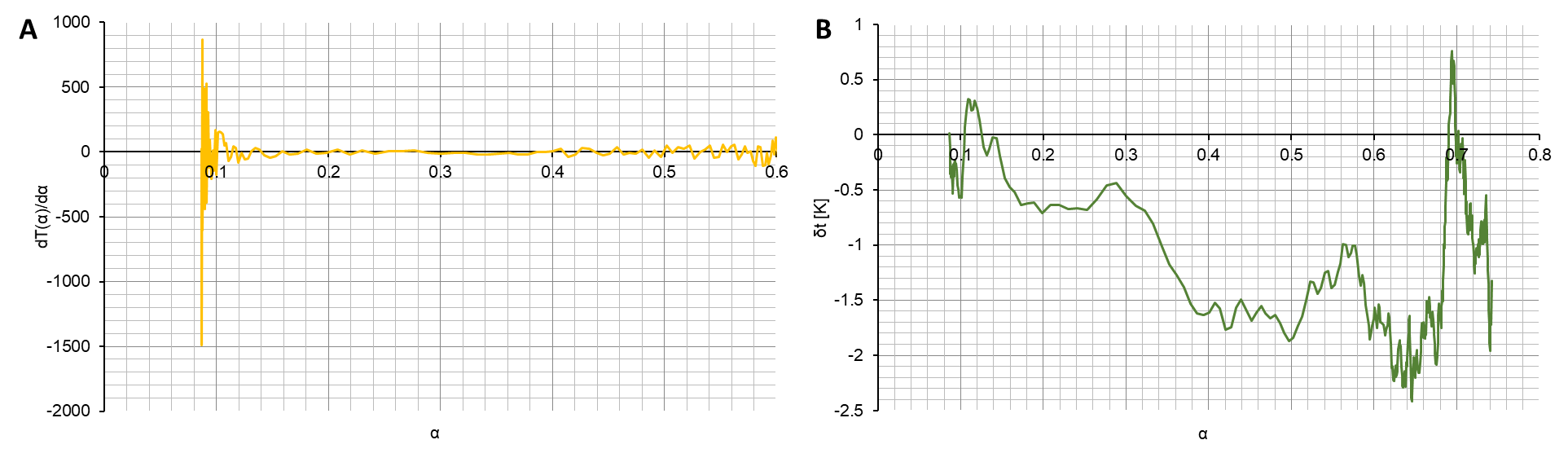
**

**Fig. S12.** Disturbance of the derivative d*T*(α)/dα with a random error of 10% (A); disturbance of temperature Δ*T* [K] determined from the disturbed derivative d*T*(α)/dα with a random error of 10%, for β=2.5.

**
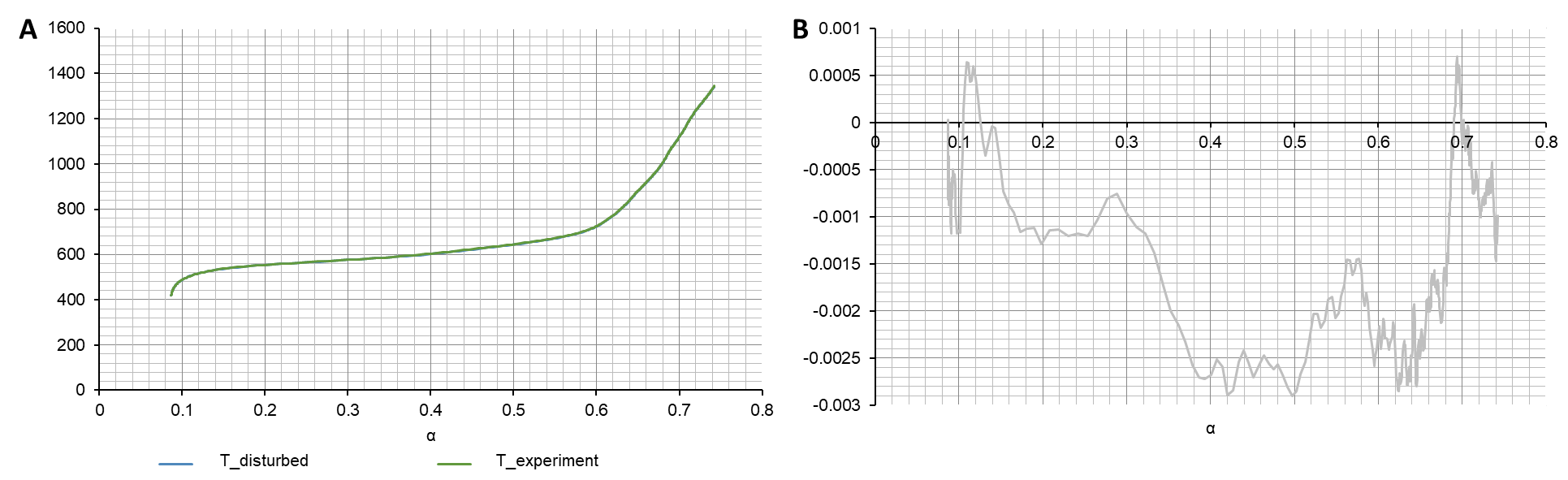
**

**Fig. S13.** Comparison of the quasi-exact temperature T_experiment_ with the temperature determined from the disturbed graph of d*T*(α)/dα with a random error of 10%, for β=2.5 (A); the margin of the relative error of disturbed temperature resulting from errors of disturbance of d*T*(α)/dα with a random error of 10%, for β=2.5.


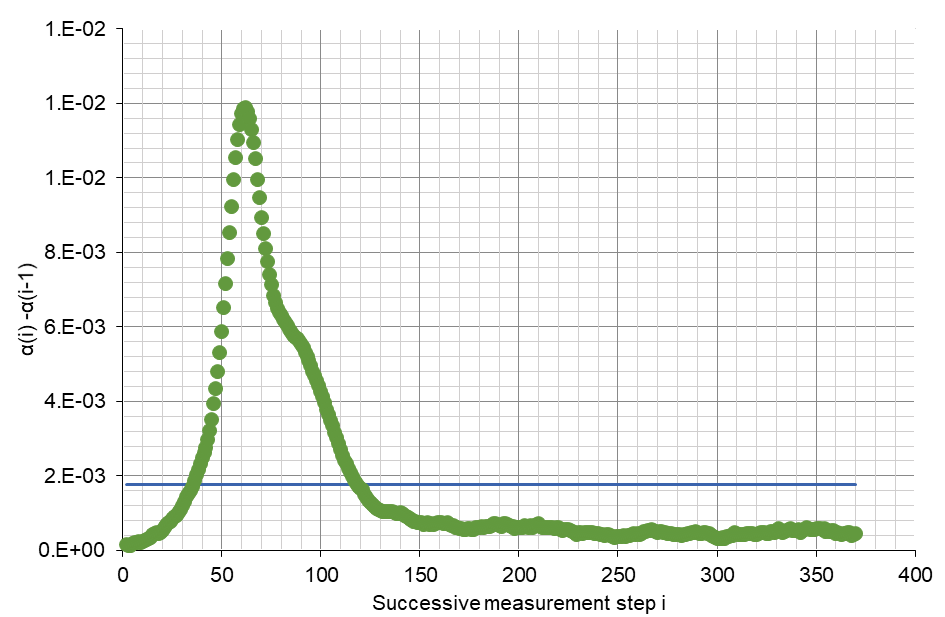


**Fig. S14**. The length of intervals α(i)–α(i-1) for successive measurement steps. Average step length αs=0.17751∙10-2 – blue line.

**Supplementary note 1 – TGA measurement**

Data used to calculations were obtained by performing thermogravimetric analysis (TG 209 Netzsch GmbH, Germany). Measurements were carried out under flowing nitrogen (10 cm^3^/min) at heating rates of 2.5, 5, 10, and 20 °C/min over a temperature range of 25–1100 °C, with an initial sample weight of approximately 7 mg.
